# Peripheral Neuropathy in Virologically Suppressed People Living with HIV: Evidence from the PIVOT Trial

**DOI:** 10.3390/v16010002

**Published:** 2023-12-19

**Authors:** Anna L. Schuldt, Henry Bern, Melanie Hart, Mark Gompels, Alan Winston, Amanda Clarke, Fabian Chen, Wolfgang Stöhr, Amanda Heslegrave, Nicholas I. Paton, Axel Petzold, Alejandro Arenas-Pinto

**Affiliations:** 1 Institute for Global Health, University College London, London WC1E 6JB, UK; 2 MRC Clinical Trials Unit at UCL, University College London, London WC1V 6LJ, UKw.stohr@ucl.ac.uk (W.S.);; 3 Queen Square Institute of Neurology, University College London, London WC1N 3BG, UK; melanie.hart@ucl.ac.uk (M.H.); a.heslegrave@ucl.ac.uk (A.H.); a.petzold@ucl.ac.uk (A.P.); 4 Service of Immunology, Southmead Hospital, Bristol BS10 5NB, UK; 5 Faculty of Medicine, Imperial College London, London SW7 2BX, UK; a.winston@imperial.ac.uk; 6 Department of HIV, Sexual Health and Contraception, Brighton and Sussex University Hospitals NHS Trust, Brighton BN11 2DH, UK; amanda.clarke16@nhs.net; 7 Florey Unit Clinic for Sexual Health, Royal Berkshire Hospital, Reading RG1 5AN, UK; fabian.chen@royalberkshire.nhs.uk; 8 Yong Loo Lin School of Medicine, National University of Singapore, Singapore 117597, Singapore

**Keywords:** peripheral neuropathy, HIV, monotherapy, protease inhibitor, metabolic syndrome

## Abstract

The aim of this study is to identify the factors associated with peripheral neuropathy and to explore neurofilament light chain (NfL) as a biomarker for peripheral neuropathy (PN) in effectively virologically suppressed adults living with HIV. All protease inhibitor monotherapy versus ongoing triple therapy in the long-term management of HIV infection (PIVOT) trial participants with data on PN at baseline were included in the study. NfL plasma levels (pNfL) were measured in a sub-set of participants. Multivariable logistic regression was used to examine the associations of PN with potential risk factors (including age, sex, nadir CD4 cell count, history of dideoxynucleoside (d-drugs) exposure, and blood glucose levels) and NfL levels. Of the 585 participants included, 131 (22.4%) reported PN during the study period (median of 44 months). The participants were predominantly male (76.6%), White (68.2%), and virologically suppressed for a median period of 37 months (range of 20–63) before recruitment. The age at baseline was 44.3 years (standard deviation (SD) of 9.2). PN was independently associated with age (adjusted odds ratio (aOR) = 1.35, 95% CI of 1.20–1.52; additional 5 years), history of d-drugs (aOR 1.88, 95% CI of 1.12–3.16), height (aOR 1.19, 95% CI of 1.05–1.35; additional 5 cm), nadir CD4 cell count (aOR 1.10 CI of 1.00–1.20; 50 cells fewer), and metabolic syndrome (aOR 2.31, 95% CI of 1.27 4.20), but not pNfL. The excess risk for PN associated with d-drug use remains after the exposure has stopped for years, suggesting non-reversible toxicity. In people with HIV, metabolic syndrome is independently associated with PN. There was no additional value for pNfL as a screening test for peripheral neuropathy in effectively virologically suppressed adults living with HIV.

## 1. Introduction

Peripheral neuropathy is known to be the most common neurologic complication at any stage in HIV infection with 30–67% of people living with HIV being affected [1,2,3]. Predominantly distal symmetric polyneuropathy (HIV-DSP) and antiretroviral toxic neuropathy (HIV-ATN) have been described [1]. The appearance of peripheral neuropathy has been well documented in patients exposed to dideoxynucleoside agents (“d-drugs”), such as didanosine (ddI), stavudine (d4T), and zalcitabine (ddC) [1,4,5]. Furthermore, previous studies suggest an association of protease inhibitor use and peripheral neuropathy in anti-retroviral therapy (ART)-experienced patients [6]. This association might be caused by a previously described interconnection of protease inhibitor use, insulin resistance, and diabetes mellitus [7], with the latter two potentially leading to peripheral neuropathy in both people living with HIV and the general population [8,9].

With improvements in survival due to effective ART, peripheral neuropathy as a consequence of the neurotoxic effects of antiretroviral drugs is more prevalent in people living with HIV at present [2]. To address this issue, the discontinuation of neurotoxic agents is recommended, as this may result in the remission of peripheral neuropathy [1].

Furthermore, irrespective of its cause, peripheral neuropathy has been found to be associated with a lower quality of life [10], which highlights the necessity for HIV neuropathy management [11]. However, effective management strategies, particularly for painful HIV peripheral neuropathy, remain mostly an unmet need. Risk factors for peripheral neuropathy in patients with uncontrolled viremia have been described, but contemporary data on people living with HIV with an effectively suppressed viral load are limited.

The aim of this study is to identify factors associated with peripheral neuropathy in adults living with HIV with an effectively suppressed viral load and to explore the levels of plasma neurofilament light chain (pNfL) as a potential biomarker for peripheral neuropathy in people living with HIV in a sub-set of the study participants. The sub-set was recruited from five of the larger PIVOT sites and participation was offered to all participants attending the final PIVOT study visit to explore neurocognitive function and neuroimaging markers. 

## 2. Methods

### 2.1. Study Design and Patients

The design and main results of the PIVOT trial have been reported elsewhere [12]; in short, it was a non-inferiority, randomized parallel-group trial (ISRCTN-04857074), conducted in 43 sites in the United Kingdom between 2008 and 2013. As part of this study, 587 virologically suppressed adults living with HIV (viral load of <50 copies/mL) for at least 24 weeks prior to entering this study were randomly assigned 1:1 to maintain ongoing triple therapy (OT) or to switch to ritonavir-boosted protease inhibitor monotherapy.

The primary outcome was the loss of future drug options, defined as new mutations with intermediate/high-level resistance to drugs in contemporary use, to which the patient’s virus was considered sensitive at trial entry. The study confirmed non-inferiority of protease inhibitor monotherapy and preserved future treatment options compared with the standard triple-drug combination ART [12]. 

Peripheral neuropathy was tested at baseline, annually thereafter, and at the final PIVOT study visit. The AIDS Clinical Trials Group (ACTG) Brief Peripheral Neuropathy Screening Tool (BPNST) was used to assess the symptoms of neuropathy, the evaluation of vibration sense and ankle jerks using a numeric rating scale (NRS), as well as a clinical examination [13]. The ACTG-BPNST has been validated against objective measures with a high specificity (89.5%) for the diagnosis of distal sensory polyneuropathy [14,15]. In brief, the presence and severity of neuropathic symptoms (i.e., pain, aching or burning, pins and needles, or numbness) was investigated. In addition, an examination was conducted to explore ankle reflexes and the perception of vibrations for over 10 s using a 128 Hz tuning fork on the big toe. Peripheral neuropathy was clinically defined as the presence of bilateral neuropathic symptoms as at least one symptom of the list above and abnormal signs, such as either reduced or absent ankle reflex or reduced vibration sense of the big toe. 

Metabolic syndrome was defined according to the International Diabetes Federation (IDF) definition as central obesity plus two or more of the following four factors: (1) elevated concentration of triglycerides or specific treatment for this lipid abnormality; (2) reduced concentration of HDL cholesterol or specific treatment for this lipid abnormality; (3) raised blood pressure or the treatment of previously diagnosed hypertension; and (4) raised fasting plasma glucose concentration or previously diagnosed type 2 diabetes [16].

This analysis included all PIVOT participants with the available data on peripheral neuropathy assessment at baseline (N = 585). Patients that fulfilled the ACTG-BPNST criteria for the presence of peripheral neuropathy at least once throughout the trial were included in the group for patients with peripheral neuropathy. 

The PIVOT neurocognitive sub-study was conducted at five of the larger PIVOT sites and was offered to all participants attending the final PIVOT study visit [17]. Of the 146 participants recruited into the sub-study, 77 had pNfL measured in stored samples collected at baseline; at weeks 48, 96, and 144; and at the final visit for this analysis.

The main PIVOT protocol and all sub-studies were approved by the Cambridgeshire 4 Research Ethics Committee and all relevant R&D offices of the National Health System (NHS). All participants provided written consent. 

### 2.2. Statistical Analysis

The variables potentially associated with peripheral neuropathy included demographics (age, sex, and ethnicity), treatment group allocation, HIV-RNA level, current and nadir CD4 cell count, height and weight, co-morbidities (history of diabetes, metabolic syndrome, blood pressure, baseline waist circumference, and fasting blood triglycerides, HDL-cholesterol, and glucose levels), and anytime exposure to d-drugs (ddI, d4T, and ddC) were investigated. Frequencies and proportions were tabulated for categorical variables and Fisher’s exact test was used for comparison; continuous variables are presented with the mean and standard deviation (median and interquartile range reported for skewed data) and were compared using either a t-test or Wilcoxon’s rank-sum test, as appropriate.

A multivariable logistic regression analysis was used to examine the association between these potential risk factors at baseline and peripheral neuropathy. All possible complete case multivariable logistic regression models were considered, and model selection was based on Akaike Information Criteria (AIC), selecting the model that yielded the lowest AIC. Unadjusted and adjusted odds ratios and associated 95% confidence intervals and *p*-values are presented for the best-fit model. The analysis was then repeated in the neurocognitive sub-study population to additionally explore the association between peripheral neuropathy and mean pNfL levels. Stata version 17 (Stata Corp LLC, College Station, TX, USA) was used for the statistical analysis.

## 3. Results

### 3.1. Study Population

Of the 587 PIVOT participants, 585 had adequately measured and reported peripheral neuropathy assessment results at baseline and were included in this analysis. A total of 131 participants (22.4%) reported symptomatic peripheral neuropathy at least once during the study period. A total of 48 of the 131 participants first reported peripheral neuropathy symptoms at baseline, with 23 of them at week 48, 27 at week 96, 15 at week 144, and 11 at the final visit. The mean age at baseline was 48.2 (±10.4) years in the group with peripheral neuropathy compared to 43.1 (±8.5) years in the group without it (*p* < 0.001). In the group presenting with peripheral neuropathy, the median number of years on ART at study entry was 4.6 (interquartile range (IQR) of 2.6, 8.1) compared to 3.9 (IQR of 2.2, 6.3) in the group without peripheral neuropathy (*p* = 0.008). None of the study participants were taking d-drugs after study entry, but 89 (15.2%) had received them in the past for a median period of 2.4 years (IQR of 0.9–4.4). The participants with past exposure to d-drugs had been off these for a median of 5.0 years (IQR of 3.4–7.3) by the time of entering in the PIVOT trial. The participants with peripheral neuropathy were more likely to have received d-drugs in the past when compared with those without it (*p* = 0.004). Other significant baseline characteristics more frequently seen in participants presenting peripheral neuropathy included larger height (*p* = 0.048), lower nadir CD4 cell count (*p* = 0.005), and metabolic syndrome (*p* = 0.001). We observed no difference in the peripheral neuropathy status according to the randomization arm (Table 1). Overall, 77.1% of the study population sustained virological suppression throughout follow-up, with no difference between participants with and without peripheral neuropathy (*p* = 0.555). Furthermore, elevated average fasting glucose levels were more common in the group reporting peripheral neuropathy when compared to the group with no peripheral neuropathy during the study period (*p* = 0.004). There was no significant difference in the characteristics when comparing reported peripheral neuropathy at baseline with the report of peripheral neuropathy at any given point in time during follow-up. 

### 3.2. Factors Associated with Peripheral Neuropathy

All variables potentially associated with neuropathic symptoms were considered within the multivariable logistic regression analysis. All possible complete case logistic regression models were compared, selecting the best-fit model according to AIC.

The final multivariable logistic regression model presented details of independent associations between the presentation of peripheral neuropathy and age at baseline visit (aOR = 1.35, 95% CI from 1.20 to 1.52, *p* =< 0.001; additional 5 years), being of Black ethnicity (aOR 1.64, 95% CI from 0.99 to 2.73, *p* = 0.055), nadir CD4 cell count (aOR = 1.10, 95% CI from 1.00 to 1.20, *p* = 0.051; 50 fewer cells), history of d-drug exposure (aOR = 1.88, 95% CI from 1.12 to 3.16, *p* = 0.017), height (aOR = 1.19, 95% CI from 1.05 to 1.35, *p* = 0.007; additional 5 cm), and metabolic syndrome at baseline (aOR = 2.31, 95% CI from 1.27 to 4.20, *p* = 0.006) (Table 2).

### 3.3. Additional Findings in the Neurocognitive Sub-Study 

Of the sub-study participants, 78 underwent imaging investigations. A total of 77 of these participants had peripheral neuropathy data at baseline and were included in this analysis (Table 3). The sub-study participants were also predominantly male (84.4%), White (83.1%), and with a median age of 43.8 (±8.9) years, and therefore, considered representative of the main study. Peripheral neuropathy was reported in 19 (24.7%) sub-study participants. The plasma levels of NfL were measured at baseline; at weeks 48, 96, and 144; and at the final visit. The mean pNfL levels were calculated. 

Consistent with the previous findings, age at the baseline visit was significantly higher in the group reporting peripheral neuropathy (*p* = 0.001). There was some evidence suggesting higher mean pNfL levels in the participants in the sub-study reporting peripheral neuropathy compared with the participants with no peripheral neuropathy (*p* = 0.0029). Whilst similar patterns were also observed for the history of d-drug exposure, nadir CD4 cell count, metabolic syndrome, and the number of years on ART prior to study entry, due to the small number of the sub-study participants and therefore an underpowered analysis, none of these differences were statistically significant in the regression models. 

When restricting the multivariable logistic regression analysis to the neurocognitive sub-study population, there remains a significant association between peripheral neuropathy and age at the baseline visit (aOR = 1.73, 95% CI from 1.21 to 2.47, *p* = 0.002; additional 5 years). 

## 4. Discussion

Peripheral neuropathy is the most common neurological complication in people living with HIV [1,2,3], with a high burden of disease for those affected [18]. It is associated with a lower quality of life [10], can affect productivity, reduces the likelihood of employment, and is associated with substantial financial costs [14,19,20]. The proportion of patients reporting peripheral neuropathy (22.4%) in this study according to the criteria of the ACTG-BPNST was comparable to that of previous studies [11]. In a systematic review by Ghosh et al., it was estimated that peripheral neuropathy amongst ART-exposed patients occurs in the range of 20.3–66% [14].

Amongst the PIVOT participants, metabolic syndrome at baseline was identified as a significant factor associated with the peripheral neuropathy status. In the general population, the individual components of the metabolic syndrome have previously been associated with peripheral neuropathy [19,20]. Particularly, an association of central obesity with peripheral neuropathy was evident in people living with severe obesity and type 2 diabetes mellitus [21,22,23]. The role of central obesity in the occurrence of peripheral neuropathy in non-diabetic people living with HIV is not yet clear. An increased body mass index and hypertriglyceridemia have previously been reported as predictors of incident neuropathic pain or peripheral neuropathy in virologically suppressed people living with HIV [24,25]. The combined effect of the individual components of the metabolic syndrome on the presence of peripheral neuropathy was evident in our study. 

In our study, history of exposure to dideoxynucleoside antiretrovirals (d-drugs) at any given point in time was identified as a risk factor for the presentation of peripheral neuropathy, even if the exposure to d-drugs had been terminated years prior the assessment. This might be due to the underlying axonopathy, as nerve regeneration may be poor, suggesting non-reversible toxicity similar to that of chemotherapy associated peripheral neuropathy [26]. In contrast, Simpson et al. identified a higher prevalence for peripheral neuropathy at the initiation of treatment with d-drugs, which commonly resulted in the discontinuation of these drugs [15]. Previous reports also stated a strong link between the exposure to d-drugs and the development of neuropathy at any given point in time [27,28,29]. 

Obesity has also been associated with higher odds of developing chemotherapy-induced peripheral neuropathy [30]. A similar association might play a role in the occurrence or persistence of neuropathic symptoms in people living with HIV who have been exposed to neurotoxic antivirals and live with obesity. Our findings support evidence from other studies of an association between heavier weight and peripheral neuropathy [6,31,32]. Additionally, we were able to identify age as a risk factor for peripheral neuropathy in accordance with previous scientific evidence [14,28,31,33]. There was also evidence suggesting an association of nadir CD4 cell count at baseline and peripheral neuropathy (*p* = 0.005). 

The pathogenesis of HIV-ATN has been linked to mitochondrial dysfunction [34], but the cause for HIV-DSP is still not fully known. A potential explanation might be the upregulation of proinflammatory cytokines and the macrophage infiltration that may result in the distal degeneration of long axons [35], but according to Mogello et al., no biomarkers of HIV severity have been identified as being associated with peripheral neuropathy in the era of effectively suppressive anti-retroviral therapy [36]. However, the necessity to identify non-invasive biomarkers for peripheral nerve damage has been highlighted in previous studies [37]. Neurofilament light chain (NfL), as a structural component of myelinated axons, has already been considered as an indicator of axonal injury in several neurodegenerative conditions [38,39]. Furthermore, there has been evidence of elevated NfL levels in the cerebrospinal fluid (CSF) of people living with HIV and associated neurocognitive impairment [40]. A study conducted by Damian et al. showed a positive association between neuropathy severity and both plasma and CSF NfL in their cohort of patients with HIV [41]. Their analysis suggested that NfL levels may be useful to evaluate active injury not only in the central nervous system, but also in peripheral nerves. However, due to the small sample size, these findings did not reach significance [41]. The neurocognitive sub-study population of the PIVOT trial was representative of the entire study population. pNfL levels were measured in this sub-study-group. We therefore explored a potential association of pNfL levels with peripheral neuropathy status in our study population. There was evidence suggesting elevated pNfL levels in patients reporting peripheral neuropathy, but this elevation was not significant in our multivariate analysis. The small effect size of blood NfL levels in most peripheral neuropathies studied is a recognized problem [39]. The small effect size was relevant because the sub-study was underpowered to demonstrate the usefulness of NfL levels as a marker for neuronal degeneration in people living with HIV. Future research should assess NfL levels associated with peripheral neuropathy in successfully virologically suppressed people living with HIV as a study conducted by Alagaratan et al., with a focus on central nervous system injury, suggested that these patients do not have increased concentrations of CSF and plasma NfL, which is evidence of neuro-axonal injury compared to HIV-negative individuals with a similar lifestyle [42].

The initial purpose of this analysis was to explore any difference in peripheral neuropathy status and progression in the treatment randomization arms (protease inhibitor monotherapy and triple therapy) in PIVOT participants. There was no significant difference in peripheral neuropathy status or progression in the two randomization arms, which supports the conclusion from the main PIVOT trial that there is no added risk from protease inhibitor monotherapy [12]. 

## 5. Limitations of This Study

Co-morbidities and additional medication should be considered to differentiate causality for peripheral neuropathy in people living with HIV and especially those who have been exposed to d-drugs. We did not collect data on prior exposure to medication for peripheral neuropathy, such as Amitriptyline, which could have modified the results of the BPNTS. 

The sub-study was underpowered to demonstrate the usefulness of NfL levels as a marker for neuronal degeneration in people living with HIV. Moreover, this analysis focused on mean pNfL levels. There was no longitudinal analysis performed on NfL levels in the sub-study group. 

## 6. Conclusions

As the most common neurological complication in people living with HIV, peripheral neuropathy is of high relevance. In accordance with previous reports, an association of age and previous exposure to d-drugs, which are no longer regularly used, was observed in our study. Additionally, an association of metabolic syndrome with peripheral neuropathy was noticeable. This underlines the importance of considering not only exposure to neurotoxic drugs but also presence of metabolic disorders when investigating neurological complaints in people living with HIV. Furthermore, our findings highlight the need to identify and manage metabolic complications in a time of increased population obesity levels. Our findings do not suggest an additional value in measuring blood NfL levels, similar to what has been observed for other chronic peripheral neuropathies.

Further studies on the specific pathogenesis of distal sensory polyneuropathy in addition to the identification of non-invasive biomarkers for peripheral neuropathy status and progression as well as a potential interaction of neurotoxic antiretroviral drugs and obesity are needed to develop more effective means to prevent and treat peripheral neuropathy in people living with HIV in the future.

## Figures and Tables

**Table 1:** Baseline characteristics according to peripheral neuropathy status—study population. Bold: Significant findings.

	No Peripheral Neuropathy Symptoms	Peripheral Neuropathy Symptoms	Overall	*p*-Value
Age at baseline				
Mean (SD)	43.1 (8.5)	48.2 (10.4)	44.3 (9.2)	**<0.001**
Sex, N (%)				
Male	345 (76.0)	103 (78.6)	448 (76.6)	0.560
Female	109 (24.0)	28 (21.4)	137 (23.4)	
Ethnicity, N (%)				
White	312 (68.7)	87 (66.4)	399 (68.2)	0.419
Black	122 (26.9)	41 (31.3)	163 (27.9)	
Other	20 (4.4)	3 (2.3)	23 (3.9)	
Allocated regimen, N (%)				
Ongoing triple therapy	226 (49.8)	64 (48.9)	290 (49.6)	0.921
Protease inhibitor monotherapy	228 (50.2)	67 (51.1)	295 (50.4)	
Years on anti-retroviral therapy (ART)				
Median (IQR)	3.9 (2.2, 6.3)	4.6 (2.6, 8.1)	4.0 (2.2, 6.7)	**0.008**
Nadir CD4 cell count				
Median (IQR)	183 (91, 251)	154 (50, 231)	178 (86, 250)	**0.005**
Baseline CD4 cell count				
Mean (SD)	553 (218)	542 (228)	551 (220)	0.611
Ever on any d-drug, N (%)				
No	396 (87.2)	100 (76.3)	496 (84.8)	**0.004**
Yes	58 (12.8)	31 (23.7)	89 (15.2)	
Baseline height (cm)				
Mean (SD)	172.9 (9.6)	174.8 (9.0)	173.3 (9.5)	**0.048**
Metabolic Syndrome—Baseline, N (%)				
No	420 (92.5)	107 (81.7)	527 (90.1)	**0.001**
Yes	34 (7.5)	24 (18.3)	58 (9.9)	

**Table 2:** Multivariable logistic regression analysis—study population. Bold: Significant findings.

	Category	Unadjusted Odds Ratio	Unadjusted *p*-Value	Unadjusted Global *p*-Value	Adjusted Odds Ratio	Adjusted *p*-Value	Adjusted Global *p*-Value
Age at baseline—additional 5 years	-	1.35 (1.21, 1.51)	**<0.001**	-	1.35 (1.20, 1.52)	**<0.001**	-
Ethnicity	White	Baseline	-	0.386	Baseline	-	0.137
	Black	1.21 (0.79, 1.85)	0.391		1.64 (0.99, 2.73)	0.055	
	Other	0.54 (0.16, 1.85)	0.326		0.82 (0.22, 3.04)	0.770	
Nadir CD4 cell count—50 cells fewer	-	1.12 (1.02, 1.22)	**0.014**	-	1.10 (1.00, 1.20)	0.051	-
Ever on any d-drug	No	Baseline	-	-	Baseline	-	-
	Yes	2.12 (1.30, 3.45)	**0.003**		1.88 (1.12, 3.16)	**0.017**	
Height—additional 5 cm	-	1.11 (1.00, 1.24)	**0.049**	-	1.19 (1.05, 1.35)	**0.007**	-
Metabolic syndrome—Baseline	No	Baseline	-	-	Baseline	-	-
	Yes	2.77 (1.58, 4.87)	**<0.001**		2.31 (1.27, 4.20)	**0.006**	

**Table 3:** Characteristics according to peripheral neuropathy status—NfL population (n = 77). Bold: Significant findings.

	No Peripheral Neuropathy Symptoms	Peripheral Neuropathy Symptoms	Overall	*p*-Value
Age at baseline				
Mean (SD)	41.9 (8.0)	49.6 (9.2)	43.8 (8.9)	**0.001**
Sex, N (%)				
Male	49 (84.5)	16 (84.2)	65 (84.4)	1.000
Female	9 (15.5)	3 (15.8)	12 (15.6)	
Ethnicity, N (%)				
White	47 (81.0)	17 (89.5)	64 (83.1)	0.841
Black	9 (15.5)	2 (10.5)	11 (14.3)	
Other	2 (3.4)	-	2 (2.6)	
Allocated regimen, N (%)				
Ongoing triple therapy	30 (51.7)	9 (47.4)	39 (50.6)	0.796
Protease inhibitor monotherapy	28 (48.3)	10 (52.6)	38 (49.4)	
Years on ART				
Median (IQR)	3.8 (2.2, 5.8)	4.9 (3.0, 10.2)	4.0 (2.3, 6.4)	0.156
Nadir CD4 cell count				
Median (IQR)	170 (90, 250)	154 (34, 250)	165 (70, 250)	0.296
Baseline CD4 cell count				
Mean (SD)	573 (230)	557 (287)	569 (244)	0.801
Ever on any d-drug, N (%)				
No	51 (87.9)	14 (73.7)	65 (84.4)	0.157
Yes	7 (12.1)	5 (26.3)	12 (15.6)	
Baseline height (cm)				
Mean (SD)	175.6 (8.9)	175.5 (7.5)	175.6 (8.5)	0.942
Baseline NfL level				
Mean (SD)	8.1 (3.5)	10.3 (4.9)	8.6 (3.9)	0.059
Metabolic syndrome—Baseline, N (%)				
No	55 (94.8)	17 (89.5)	72 (93.5)	0.592
Yes	3 (5.2)	2 (10.5)	5 (6.5)	

## Data Availability

The PIVOT data are held at MRCCTU at UCL, which encourages the optimal use of data by employing a controlled access approach to data sharing, incorporating a transparent and robust system to review requests and provide secure data access. Anonymized individual participant data and study documents can be requested from the corresponding author and will be made available, subject to approval of the Trial Steering Committee.
